# Localized Small Bowel Adenocarcinoma Management: Evidence Summary

**DOI:** 10.3390/cancers14122892

**Published:** 2022-06-11

**Authors:** Anthony Turpin, Mehdi El Amrani, Aziz Zaanan

**Affiliations:** 1 UMR9020-UMR-S 1277 Canther-Cancer Heterogeneity, Plasticity and Resistance to Therapies, University of Lille, CNRS, Inserm, CHU Lille, Institut Pasteur de Lille, 59000 Lille, France; 2 Medical Oncology Department, CHU Lille, University of Lille, 59000 Lille, France; 3 Department of Digestive Surgery and Transplantation, Lille University Hospital, 59000 Lille, France; mehdi.elamrani@chu-lille.fr; 4 Department of Gastroenterology and Digestive Oncology, European Georges Pompidou Hospital, AP-HP Centre, 75015 Paris, France; aziz.zaanan@aphp.fr; 5 Centre de Recherche des Cordeliers, INSERM, Sorbonne Université, Université de Paris, MEPPOT, 75006 Paris, France

**Keywords:** small bowel adenocarcinoma, duodenal cancer, jejunoileal cancer, surgery, adjuvant chemotherapy, perioperative chemoradiation, biomarkers

## Abstract

**Simple Summary:**

Small bowel adenocarcinoma is a rare but aggressive disease that requires peri-operative treatment. Due to its rarity, there is little data on small bowel adenocarcinoma treatment, and most recommendations come from expert agreements or analogies to the management of colon cancer. In this Review, we summarize the current evidence on the management of localized small bowel adenocarcinoma, including future biomarker research perspectives that may help identify high-risk small bowel adenocarcinomas.

**Abstract:**

Small bowel cancers are rare diseases whose prognosis is poorer than that of colon cancers. Due to disease rarity, there is little data on small bowel adenocarcinoma (SBA) treatment, and most recommendations come from expert agreements or analogies to the management of colon cancer. Although relatively high rates of local recurrence are observed for duodenal malignancies, distant metastatic relapse remains common and requires adjuvant systemic therapy. Given the similarities between SBA and colorectal cancer, radiotherapy and chemotherapy strategies used for the latter disease are frequently pursued for the former disease, specifically for tumors located in the duodenum. However, no previous randomized study has evaluated the benefit of adjuvant chemotherapy on the overall survival of SBA patients. Most previous studies on treatment outcomes and prognostic factors in this context were based on large international databases, such as the Surveillance, Epidemiology, and End Results or the National Cancer Database. Studies are required to establish and validate prognostic and predictive markers relevant in this context to inform the use of (neo) adjuvant treatment. Among those, deficient mismatch repair tumors represent 20% of SBAs, but their impact on chemosensitivity remains unknown. Herein, we summarize the current evidence on the management of localized SBA, including future perspectives.

## 1. Introduction

Small bowel cancers are rare diseases, accounting for approximately 5% of gastrointestinal cancers (Siegel, 2020) [1], with the predominance of small bowel adenocarcinoma (SBA), most commonly located in the duodenum (Locher, 2018) [2].

Studies based on the French prospective clinico-biological database (called NADEGE cohort) have shown that most patients have localized disease at diagnosis and that the associated prognosis is worse than that in colon cancers (Aparicio, 2020) [3]; this finding is consistent with that of a previous study (Howe, 1999) [4]. After a median follow-up of 54 months, the 5-year overall survival rate was 87.9%, 78.2% and 55.5% for disease stages I, II and III, respectively (Aparicio, 2020) [3].

Predisposing diseases are familial adenomatous polyposis, Lynch syndrome, Peutz-Jeghers syndrome, Crohn’s disease and celiac disease (Green PH 2002) [5]. In the NADEGE cohort, the prevalences of an associated predisposing disease were 8.7%, 6.9%, 1.7%, 1.7% and 0.6% for Crohn’s disease, Lynch syndrome, familial adenomatous polyposis, celiac disease and Peutz-Jeghers syndrome, respectively (Aparicio T., 2020) [3].

Given its rarity, there is little evidence on the treatment of SBA; in fact, most recommendations come from expert agreements or from analogies to the management of colorectal cancer (CRC), requiring multidisciplinary discussions per case (Locher, 2018) [2]. Most studies on the treatment of localized SBA are retrospective or derived from large databases such as the National Cancer Database or Surveillance, Epidemiology, and End Results (SEER) database. These large retrospective epidemiological databases account for tumor location (duodenum, jejunum and ileum); however, recent studies have shown that tumors that share a location remain heterogenous and that each tumor is associated with a different prognosis and requires a tailored approach to therapy, including surgery (Chow, 1996; Howe, 1999; Gustafson, 2008) [4,6,7]. Herein, we aim to summarize the present evidence on the treatment of localized SBA, including future perspectives.

## 2. Surgical Approaches

According to international guidelines, surgical resection of SBA requires a thorough exploration of the abdominal cavity due to the high risk of peritoneal invasion. Surgical treatment is based on the principle of a monobloc resection of the tumor with a distal and proximal margin of at least 5 cm. It also requires a healthy circumferential margin and a monobloc removal of the adjacent mesentery with the localization of the vascular pedicle (distal lymph nodes) and an adequate locoregional lymph node dissection (Locher, 2018; Benson, 2019) [2,8].

In contrast to pancreatic ductal adenocarcinomas, which diffusely infiltrate the surrounding soft tissues, the extension of duodenal adenocarcinomas into adjacent tissues is usually localized, and tumor-free resection margins can be obtained without resecting adjacent organs or soft tissues (Sohn, 1998; Brücher, 2001; Abrahams, 2002) [9,10,11].

Technically, resection of the primary and investing mesentery allows the removal of both primary cancer and regional nodes at risk of metastasis and provides important information for staging. However, adequate mesenteric resection may be limited by the proximity of local lymph nodes or the location of a primary tumor within the superior mesenteric artery. The optimal number of regional lymph nodes required for adequate staging is subject to debate (Overman, 2010; Overman, 2012; Tran, 2015; Wilhem, 2016) [12,13,14,15]. However, the National Comprehensive Cancer Network (NCCN) guidelines recommend the use of at least eight regional nodes (Benson, 2019) [8].

The type of resection depends on tumor location (Locher, 2018; Benson, 2019) [2,8]. For localized cancers of the jejunum or ileum, segmental bowel resection with localized lymph node dissection is often performed. The anatomical proximity of the duodenum to the cephalic pancreas makes the surgical management of duodenal cancers different from that of cancers at other intestinal locations. 

Cephalic duodeno-pancreatectomy (CDP) is required for tumors involving the second portion of the duodenum and for those that invade the ampulla of Vater or the pancreas. For tumors involving the first, third and fourth portions of the duodenum that do not involve the pancreas or the ampulla of Vater, the evidence on the need for CDP versus segmental duodenal resection remains controversial. A recent study of 1611 duodenal cancer patients showed that radical resection by CDP was not associated with improved prognosis compared to segmental duodenal resection (Cloyd, 2015; Platoff, 2020) [16,17]. In contrast, lymph node staging improved after radical resection by CDP. A total of eight studies (Kaklamanos, 2000; Tocchi, 2003; Kelsey, 2007; Han, 2008; Cecchini, 2012; Onkendi, 2012; Cloyd, 2015; Jiang, 2016) [16,18,19,20,21,22,23,24] comparing the survival of patients treated with CDP or segmental resection were included in a meta-analysis. These studies reported no significant difference in survival when comparing outcomes of segmental resection to those of CDP (Meijer, 2018) [25]. Two studies reported more overall and more positive lymph nodes removed with CDP than with segmental resection (Cloyd, 2015; Onkendi, 2012) [16,23]. However, these findings are inconsistent with those of another study, which showed no survival differences (Kaklamanos, 2000) [18].

Overall, most studies, despite small samples, show similar results for CDP and segmental resection. A recent study has demonstrated better recurrence-free survival (RFS) (39 months vs. 13 months) after CDP than after segmental resection (Colina, 2020) [26].

Moreover, SBA is often associated with several predisposing conditions: Crohn’s disease, celiac disease, Lynch Syndrome and familial adenomatous polyposis, among others. However, while these predisposing conditions may have implications in terms of screening for patients who have not yet developed small bowel cancer, there are no veritable implications in terms of surgical or adjuvant procedures. In particular, due to a lack of literature, there is no indication for prophylactic surgery for small bowel cancers.

## 3. Medical Approaches

SBA relapse tends to be metastatic, with one retrospective study reporting that distant and locoregional relapse accounts for 86% and 18% of all recurrences, respectively (Dabaja, 2004) [27]. Although a higher rate of local recurrence is observed for duodenal malignancies, distant metastatic relapse remains predominant (Bakaeen, 2000) [28]. These findings suggest a need for (neo)adjuvant systemic therapy.

### 3.1. Neoadjuvant Chemo-Radiotherapy

According to international guidelines (Locher, 2018; Benson, 2019) [2,8], in the absence of distant metastasis, primary surgery is indicated for resectable localized tumors unless posterior invasion impairs R0 tumor resection. In this case, preoperative treatment may be required to make the lesion resectable (expert opinion). For example, in one study, 9% of patients were offered neoadjuvant treatment of heterogenous modalities, with radiation therapy delivered in 14% of the cases (Colina, 2020) [26].

There is limited evidence to support the role of neoadjuvant chemotherapy or chemo-radiotherapy (CRT) in locally advanced SBA. In a study based on the National Cancer Database, neoadjuvant chemotherapy was associated with better overall survival in the “proximal” (duodenum) cohort (*p* < 0.01), while adjuvant chemotherapy was associated with better overall survival in the “proximal” (*p* < 0.01) and “distal” (jejuno-ileal) cohorts (*p* < 0.01), compared to surgery alone. These results may be due to differences in biological and pathological characteristics between duodenal and jejuno-ileal tumors, as proximal tumors are more likely than distant tumors to have a higher grade (Tiffany C. Lee, 2020) [29]. A meta-analysis of five studies (*n* = 117) revealed no impact of preoperative chemotherapy or CRT on overall survival (Meijer, 2018) [25]. Kelsey et al. evaluated neoadjuvant CRT and surgical outcomes in a case series of locally advanced duodenal adenocarcinoma. Two (18%) patients had a complete pathological response (Kelsey, 2007) [20]. Meanwhile, all patients treated with surgery alone had invasive lymph nodes postoperatively, whereas none of the patients who received preoperative CRT had pathological lymph node positivity, suggesting the potential locoregional downstaging benefits of CRT (Kelsey, 2007) [20]. A separate study has shown that, among 10 patients with locally advanced disease, most (90%) initially unresectable tumors became resectable tumors (Onkendi, 2012) [30].

### 3.2. Adjuvant Procedures

#### 3.2.1. Adjuvant Chemotherapy

The NCCN guidelines recommend adjuvant chemotherapy after surgery for SBA stages II and III (Benson, 2019) [8]. However, no randomized study has evaluated the benefit of this approach to overall survival (de Bree, 2018) [31]. The benefit of adjuvant therapy remains subject to debate after several small retrospective single-center studies showed some benefits of adjuvant therapy for high-risk patients. However, these findings are likely subject to selection bias; a need for varied chemotherapy regimens is likely in this context (de Bree, 2018) [31].

A retrospective study of 241 patients with resected SBA (stages I–III) treated over 22 years revealed that 35% of the patients received adjuvant chemotherapy. Among those treated with this modality for stage III SBA, the median overall survival was 33.8 months, compared with 24.7 months in patients treated without it (*p* < 0.01) (Huffmann, 2020) [32]. No benefit was demonstrated in patients with stage I or II diseases. FOLFOX and 5FU were provided to most patients. Other less commonly used treatments included capecitabine/oxaliplatin, capecitabine alone and irinotecan alone. Compared to no therapy at all, FOLFOX was associated with improved overall survival in patients with stage III disease (*p* = 0.02) (Huffmann, 2020) [32].

Furthermore, a retrospective multicenter study of the Asian population revealed that “combined” adjuvant chemotherapy was independently associated with disease-free (*p* = 0.002) and overall survival (*p* = 0.001). Monotherapy was not superior to surgery alone in terms of overall survival (26.5 vs. 26.0 months, respectively) (Li, 2020) [33]. Meanwhile, Overman reported that adjuvant therapy affected disease-free (*p* = 0.05) but not overall (*p* = 0.23) (*n* = 54) survival. Nevertheless, the impact of adjuvant regimens was associated with the modalities they were combined with, including radiation therapy, chemotherapy, and chemoradiation. The outcomes of patients treated with adjuvant chemotherapy alone (*n* = 18) were compared to those of patients who received no adjuvant therapy, revealing no impact of adjuvant chemotherapy on either disease-free (*p* = 0.11) or overall (*p* = 0.36) survival [34] (Overman, 2010). Meanwhile, the National Cancer Database studies showed overall survival benefits for proximal (duodenum; *p* < 0.01) and distal (jejunum or ileum; *p* < 0.01) tumors (Lee, 2020; Eckert, 2016) [29,35] (Table 1).

A meta-analysis of 26 studies (*n* = 6438) on duodenal cancer of any stage failed to show any survival benefit of adjuvant chemotherapy. In five studies that involved tumor resection, the pooled 5-year overall survival rates were comparable between groups that received adjuvant chemotherapy (*n* = 263) and those treated with surgery alone (*n* = 148) (48% vs. 46%, respectively, *p* = 0.70) (Meijer, 2018) [25]. In this study, 98% of patients receiving adjuvant chemotherapy were treated with an intravenous or oral fluorouracil-based regimen, either as monotherapy or in combination with platinum salts (Meijer, 2018) [25]. However, these findings should be approached with caution because different chemotherapy regimens were used, and the analysis was not stratified. Furthermore, most patients undergoing adjuvant treatment (74%) received adjuvant radiotherapy combined with chemotherapy, which precludes any adjuvant chemotherapy benefit assessment (Meijer, 2018) [25].

Another meta-analysis of 15 studies (*n* = 5986) showed no effect of adjuvant chemotherapy on survival (pooled hazard ratio (HR) = 0.89, *p* = 0.25) (Ye, 2018) [42]. Similar results were reported for 607 duodenal adenocarcinoma patients (pooled HR = 0.96, *p* = 0.77). Recurrence rates were comparable between the groups treated with and without adjuvant chemotherapy (pooled HR = 0.89, *p* = 0.48) (Ye, 2018) [42]. Nevertheless, the uptake of adjuvant chemotherapy has increased from 8% in 1985 to 24% in 2005 (Bilimoria, 2009) [43] and from 24.2% in 1998 to 43.4% in 2011, according to the National Cancer Database (Ecker, 2016) [35]. FOLFOX and CAPOX are the most common regimens, based on adjuvant colon cancer treatment recommendations and findings from phase II studies on metastatic SBA (Overman, 2009; Xiang, 2012; Nakayama, 2017) [44,45,46]. 

Among patients included in the prospective French NADEGE database, most patients treated between 2009 and 2012 received adjuvant chemotherapy for localized SBA, accounting for 61.5% of this patient group, including 46.3% and 84.6% of stage II and III disease cases. The oxaliplatin-based doublet chemotherapy, FOLFOX or XELOX, was the most frequent adjuvant regimen (89.9%) in contrast to fluoropyrimidine monotherapy (9.1%) (Aparicio, 2020) [3]. However, 3-year overall survival rates for stage III SBA patients were comparable to those treated with and without adjuvant chemotherapy (69.9% vs. 69.2%, respectively; *p* = 0.9496) (Aparicio, 2020) [3]. In the NADEGE cohort, patients were not randomized regarding the use of chemotherapy, which may have affected the results [3].

Finally, another study based on the National Cancer Database comparing outcomes of stage III SBA patients treated with adjuvant chemotherapy (*n* = 1142) with those of patients treated with surgery alone (*n* = 1155) revealed a significant decrease in the risk of death in the former compared to the latter group (median overall survival 42.4 vs. 26.1 months; *p* < 0.001) using propensity score matching analysis (Ecker, 2017) [35]. Some overall survival benefits associated with adjuvant chemotherapy were also observed without any significant differences in patients with stage I (158 vs. 110 months, *p* = 0.226) and II (104 vs. 79 months, *p* = 0.185), respectively (Ecker, 2016) [35].

The international randomized phase III benefit of adjuvant chemotherapy for SBA (BALLAD) trial (NCT02502370), evaluating the benefit of adjuvant chemotherapy after curative R0 surgery in stage I (excluding T1aN0), II, or III SBA is currently on-going. Patients were randomized to undergo surgery alone or surgery combined with adjuvant chemotherapy with either LV5FU2 or FOLFOX. In parallel, the CAPOX regimen is being evaluated in Japanese patients in the phase III J-BALLAD trial (UMIN000027280) conducted with the same methodology but in the Asian population (Kitahara, 2019) [47]. These trials will provide the first prospective results on the effect of adjuvant chemotherapy in localized SBA.

#### 3.2.2. Adjuvant Chemo-Radiotherapy

Given the proximity to the pancreas, patients with SBA in the duodenum are often recommended interchangeable radiotherapy and chemotherapy strategies. A previous study reported no differences in overall survival in patients treated with adjuvant chemo-radiotherapy or chemotherapy (48.9 vs. 43.5 months, *p* = 0.669). Chemo-radiotherapy was not associated with survival benefits after positive margin surgical resection (*n* = 133; 27.6 vs. 18.5 months; *p* = 0.210) or in cases with T4 tumor stage (*n* = 461; 30.6 vs. 30.4 months, *p* = 0.844), inadequate lymph node removal (*n* = 584; 40.5 vs. 43.2 months, *p* = 0.707), lymph node positivity (*n* = 647; 38.3 vs. 34.1 months, *p* = 0.622), or poorly differentiated tumors (*n* = 429; 46.6 vs. 35.7 months, *p* = 0.434) (Eckert, 2017) [48]. 

The feasibility of adjuvant radiotherapy in SBA was shown in a retrospective study of 24 patients that underwent surgery for duodenal cancer by CDP. Patients treated with adjuvant chemo-radiotherapy tended to have better locoregional relapse-free survival than their counterparts (*p* = 0.07). No patient experienced grade 3 or higher toxicity during irradiation (Kim, 2012) [49]. Other studies have failed to demonstrate the survival benefits of chemo-radiotherapy versus surgery alone (Bakaeen, 2000; Kelsey, 2007; Poultsides, 2012) [20,28,50]. In fact, a previous study reported equivalent 5-year survival rates (47% vs. 48%) in patients treated with adjuvant chemo-radiotherapy and those treated with surgery alone, despite a higher rate of positive lymph nodes in patients treated with the former method than in those treated with the latter method (Poultsides, 2012) [50]. In a separate study, patients that received preoperative or postoperative chemo-radiotherapy had overall survival rates comparable to those of patients treated with surgery alone; however, pathological outcomes in the chemo-radiotherapy groups were less favorable than those in other groups (Kelsey, 2007) [20] (Table 2). 

In a larger study based on the National Cancer Database, outcomes associated with adjuvant chemo-radiotherapy were compared with those associated with adjuvant chemotherapy (*n* = 1028), revealing comparable survival rates in matched analysis, even in the case of pejorative histoprognostic criteria (Eckert, 2017) [48]. Furthermore, a meta-analysis of studies on adjuvant chemo(radio)therapy did not show any survival effects after adjustment for lymph node status (Meijer, 2018) [25].

## 4. Prognostic Factors and Biomarkers for (Neo)Adjuvant Treatment

Prognostic factors were examined in retrospective studies based on the SEER and National Cancer Database datasets, and other retrospective studies of rare diseases (Overman, M.J, 2010; Colina, A; 2020, Lee, T.C. 2020, Halfdanarson, T.R, 2010, Bilimoria, 2009; Legué, L.M, 2016; Thiessen, M, 2020; Falcone, R, 2019) [12,26,29,36,43,51,52,53].

Nevertheless, given the discrepancies in survival findings from studies on adjuvant chemotherapy and the lack of relevant randomized studies, biomarker efficacy remains subject to debate. These discrepancies in the value of adjuvant chemotherapy are particularly marked for stage II patients, who account for up to 45% of SBA patients (Eckert, 2016) [35] and have 5-year cancer-specific survival rates of 55%, which are lower than those for colon cancer patients, estimated at approximately 84% (Overman, 2012) [13]. 

The French intergroup guidelines suggest adjuvant chemotherapy for stage II disease and pT4 tumors (expert agreement) (Locher, 2018) [2]. The NCCN guidelines (Benson, 2019) [8] recommend adjuvant chemotherapy for this patient group based on the criteria used for colon cancer, which include: (i) microsatellite status that may indicate chemosensitivity in proficient MMR status or chemoresistance in dMMR tumors, respectively, affecting prognosis, and (ii) a “low” number of removed nodes (without any defined cutoff), pT4 or perforated tumors, or invasive resection margins. Other factors that may be considered are high histological grade and lymphatic and peri-nerve emboli (Benson, 2019) [8]. Lymph node involvement as a prognostic factor has the strongest evidence base.

### 4.1. Lymph Node Involvement

A recent study reported 5-year survival rates for patients with stage I, II and III disease as 79%, 58% and 38%, respectively (*p* < 0.001); the corresponding Recurrence-Free Survival (RFS) rates were 80%, 40% and 26%, respectively (*p* < 0.001) (Colina, 2020) [26]. A meta-analysis of 11 studies reported 5-year survival rates that were higher for patients with node-negative than for those with node-positive tumors (65% vs. 21%; OR = 0.17, *p* < 0.0001) (Meijer, 2018) [25]. Node involvement remains an independent prognostic factor in most studies after adjusting for other clinicopathological factors, including tumor size, differentiation grade and disease stage (Meijer, 2018). Meanwhile, in a recent meta-analysis, lymph node involvement, poorly differentiated disease, high tumor grade and invasive resection margins were significantly associated with survival (Ye, 2018) [42].

In stage III SBA, the involvement of three or more lymph nodes was associated with decreased 5-year disease-free survival rate in a SEER study (Overman, 2010) [12]. In this study, cancer mortality decreased significantly as the total number of nodes assessed increased in patients with localized disease (*p* < 0.001). The presence of more than seven lymph nodes was associated with improved cancer survival (Overman, 2010) [12].

The AJCC guidelines (8th edition) [54] (Coit DG, 2017) state that six is the minimum number of regional nodes for correct staging and effective treatment, although other studies recommend higher thresholds (Sarela, 2004; Nicholl, 2010) [55,56]. In one study, the number of operated nodes greater than seven was associated with a significant increase in RFS but not in overall survival rates (operated nodes < 7 versus ≥ 7, 31 vs. 203 months, *p* = 0.02, and 61 vs. 132 months, *p* = 0.38, respectively) (Colina, 2020) [26]. These findings suggest a need for extensive resection and high node count removal in SBA patients (Nicholl, 2010; Overman, 2010; Ecker, 2016) [12,35,55].

### 4.2. Tumor Location

A previous study has shown that “proximal” (duodenum) SBA tends to present as a higher grade, moderately to poorly differentiated disease with lymph node involvement (*p* < 0.01); in contrast, “distal” (jejuno-ileum) SBA tends to present with a larger tumor size (*p* < 0.01) and better prognosis (Lee, 2020) [29]. These findings are consistent with those of other studies (Tiffany C. lee, 2020; Eckert, 2016; Zhou, 2021) [29,35,57].

### 4.3. MMR Status

Microsatellite instability is a phenotypic consequence of the inactivation of the DNA base mismatch repair (MMR) system. This deficit in the MMR system (dMMR), found in 12–15% of colorectal cancers and in 20–30% of SBAs, is associated with a germline mutation in one of the MMR system genes (MLH1, MSH2, MSH6 or PMS2 genes; Lynch syndrome) or with hypermethylation of the promoter of the MLH1 gene (sporadic forms) (Zaaimi, 2016; Latham, 2021; Suerink, 2021) [58,59,60]. In localized SBA, the dMMR phenotype is more frequently observed in proximal than in distal tumors (Aparicio, 2013) [61].

In colon cancer, several studies have shown that the dMMR phenotype may predict good outcomes as well as 5FU resistance (Popat, 2005) [62]. For example, the French National Cancer Thesaurus does not recommend adjuvant chemotherapy for stage II dMMR colon cancers. In contrast, the combination of fluoropyrimidine with oxaliplatin remains the standard treatment for stage III colorectal cancer, regardless of MMR phenotype (Lecomte, 2021) [63]. Indeed, preliminary data suggest that the addition of oxaliplatin to 5FU may restore the efficacy of adjuvant chemotherapy for stage III dMMR colon cancers (Zaanan, 2014) [64]. 

The dMMR status, although considered beneficial in stage II SBA and associated with reduced chemosensitivity, based on colorectal cancer findings (Popat, 2005) [62], needs to be better understood in localized small bowel cancers; dMMR status may affect approximately 42% of stage II SBA patients (*n* = 66) (Vanoli, 2021) [65]. The Small Bowel Italian Consortium has reported that dMMR status and celiac disease may be associated with improved cancer-specific survival rates.

Furthermore, SBAs associated with Lynch syndrome represent 6.2–10% of localized tumors (Aparicio, 2020; Latham, 2021) [3,59]. Among 100 patients with any stage of SBA, dMMR status was associated with an earlier disease stage and lower recurrence rates (Latham, 2021) [59]. These findings are similar to those of studies on colorectal cancer, showing that dMMR tumors are more often diagnosed at a non-metastatic stage and tend to have better outcomes than other tumor types (Aparicio T., 2021) [66].

Meanwhile, proficient MMR status has been associated with a decreased risk of locoregional recurrence but with an increased risk of distant recurrence compared to the risks associated with dMMR status in univariate analysis. A follow-up multivariate analysis revealed that lymph node involvement, poor histological differentiation, “non-black” race, perineural invasion and lymphovascular invasion are independent predictors of poor survival in SBA (Colina, Open (2020) [26].

### 4.4. Other Prognostic Factors

Other prognostic factors include tumor size, differentiation grade, lymphatic and peri-nerve emboli and resection margin invasion (Table 3).

## 5. Nomograms and Risk Scores

Small retrospective studies lack the power required to establish prognostic scores applicable in clinical decision-making. However, Colina proposed seven covariates that may be considered in a multivariate model of RFS, including lymph node involvement (stage III SBA), lymphovascular invasion status, histological grade and ethnicity. A separate set of 10 covariates was evaluated in a multivariate model of overall survival, including ethnicity, stage, histological grade and perineural invasion (Colina, 2020) [26]. 

Other SEER-based studies of all-stage SBA used multivariate models to establish nomograms to predict overall and cancer-specific survival rates at 3 and 5 years. One such study included age, marital status, tumor location, grade, TNM stage and surgery status (*p* < 0.05) (Zheng, 2020) [67]. A separate study based on the same data source reported that age, gender, tumor location, TNM stage, metastatic site, surgery status, invaded lymph node count and chemotherapy status were prognostic factors in this context (Gu, 2021) [68]. These nomograms require prospective validation in homogeneous populations, excluding patients with metastatic disease.

## 6. Conclusions and Futures Directions

SBAs are rare and serious disease entities that require expert management by a multidisciplinary team and may be treated both surgically and medically. Although no prospective trial has evaluated the survival benefits of adjuvant therapy after complete resection of SBA (Aparicio T., 2014) [69], several retrospective studies reported conflicting findings.

Previous study findings should be interpreted with caution due to limitations such as small samples and heterogenous adjuvant treatments used. 

Despite conflicting evidence, the French National Cancer Thesaurus and NCCN, among others, recommend adjuvant chemotherapy with a combination of fluoropyrimidine and oxaliplatin after complete resection of stage III or IIB (T4N0) SBA (Locher, 2018, Benson, 2019) [2,8]. Nevertheless, stage IIA (T3N0) tumors have a high risk of recurrence, specifically perforated tumors or tumors with high-risk features, which may benefit from adjuvant chemotherapy with fluoropyrimidine alone or in combination with oxaliplatin. High-risk features include, in addition to the T4 stage, close or positive surgical margins, few examined lymph nodes (<5 for duodenal or <8 for jejunal/ileal primary tumor location) or tumor perforation [8] (Table 4). These recommendations are extrapolated from the recommendations for adjuvant chemotherapy in colon cancers. Although adjuvant radio-chemotherapy is unlikely to improve survival outcomes, in locally advanced SBA, it may help achieve tumor downstaging (Locher, 2018, Benson, 2019) [2,8].

Prognostic factors for SBA have been derived from retrospective or large database studies. Further studies are required to define and validate these prognostic markers before (neo)adjuvant treatments are recommended. 

In CRC, the IDEA study defined low versus high risk in stages II and III (Grothey, 2018; Iveson, 2021) to discuss indications regarding chemotherapy protocols and duration of treatment [70,71]. Studies in this direction are also necessary for the localized SBA. 

A prospective, international, randomized trial (BALLAD trial, NCT02502370) is closed to enrolment and aims to evaluate the efficacy of adjuvant chemotherapy with fluoropyrimidine monotherapy or combination therapy with FOLFOX after R0 resection of stage I-III SBA. The results of this trial are expected to be presented at a future international meeting. This prospective trial will help validate biomarkers because archival formalin fixed paraffin embedded tissue and contemporaneous venous blood samples are collected from every registered patient to allow molecular profiling and translational research.

In CRC, circulant tumoral DNA (ctDNA) may help identify residual disease post-surgery and post-adjuvant treatment (Mauri, 2022) [72]. CRC is a solid tumor that sheds a high amount of ctDNA in the bloodstream (Bettegowda, 2014; Nakamura, 2020) [73,74,75]. Given the characteristics shared with CRC, SBA may also have a high ctDNA shedding rate, which should be examined in future studies.

Finally, the impact of MMR phenotype on the efficacy of adjuvant chemotherapy in stage II and III SBA remains unknown, although preliminary data suggest dMMR may be associated with less aggressive disease and lower adjuvant chemotherapy efficacy (Latham, 2021) [59]. The results of larger international retrospective or prospective studies are required to elucidate these associations. 

Moreover, preliminary results of a phase II open-label, single-center trial (NCT04082572) of dMMR non-metastatic localized unresectable or high-risk resectable (defined as ≥20% recurrence) solid tumors treated with neoadjuvant pembrolizumab have reported an overall response rate of 77% [76]. Encouraging pathological complete response rate (higher than 50%) data provide a foundation for further studies on non-operative management of dMMR localized solid tumors. 

In this context, genomic profiling can identify potentially targetable genomic alterations in the majority of SBA cases (91%), and the higher incidence of microsatellite instability and tumor mutational burden in SBA suggests a potential role for immunotherapy (Schrock, 2017) [77], which needs to be evaluated in the adjuvant setting.

In conclusion, SBA is a rare but aggressive disease that requires perioperative treatment. Results of ongoing trials (NCT02502370) will provide evidence for novel perioperative strategies, including adjuvant chemotherapy. 

Biomarkers, such as ctDNA or MMR status, may help identify high-risk characteristics of SBAs, thus helping treatment.

## Figures and Tables

**Table 1:** Efficacy of adjuvant chemotherapy on disease-free survival and overall survival in small bowel cancers.

Author (Year)	Design	Population	Location	Stage	N (Surg/Surg + CT)	DFS (Surg vs. Surg + Adj CT)	OS
Overman (2010) [34]	Retrospective single-center	Caucasian, US	Duodenum: 67% Jejunum: 20% Ileum: 13%	I: 33% II: 38% III: 29%	54 (24/18)	No effect (*p* = 0.11)	No effect (*p* = 0.36)
Halfdanarson (2010) [36]	Retrospective medical records	Caucasian, US	Duodenum: 57%, Jejunum: 29% Ileum: 10%	I: 8% II: 29% III: 28% IV: 35%	491 (ND/34)	N/A	No effect (*p* = 0.44)
Dong Hoe Koo (2011) [37]	Retrospective	Asian, Korea	Duodenum: 65.4% Jejuno-ileum: 36.4%.	I: 15.4% II: 38.2% III: 46.2%	52 (29/23)	No effect HR 1.40; 95% CI, 0.50–3.94	No effect HR 0.80; 95% CI, 0.31–2.07
Inoue (2012) [38]	Retrospective single-center	Asian, Japan	Duodenum: 66.7% Jejuno-ileum: 33.3%	I–II: 56% III–IV: 44%	25 (13/12)	N/A	No effect (*p* = 0.055, univariate)
Khurum Khan (2015) [39]	Retrospective single-center	Caucasian, UK	Duodenum: 62.5% Jejunum: 20.8% Ileum: 14.6 NS: 2.1%	I/II: 62.5% III: 25% ND:12.5%	48 (48/27)	Median relapse-free survival: 31.1 months (95% CI: 8.0–54.3).	Median OS: 42.9 months
Donat Duerr (2016) [40]	Retrospective single-center	Caucasian Swiss/Canada	Duodenum: 48% Jejunum: 31% Ileum: 21%	I: 6% II: 27% III: 21% IV: 37% ND: 9%	76 (49/27)	No effect (*p* = 1)	No effect (*p* = 0.211)
Ecker (2016) [35]	National Cancer database	Caucasian, US	Duodenum 36% Jejuno-ileum: 43% NS: 21%	I: 3% II: 43.7% III: 53.3%	2297 (1155/1142)	N/A	Significant improvement median OS, 63.2 vs. 44.5 months (*p* < 0.001)
Aydin (2017) [41]	Retrospective	Turkey	Duodenum 70% Jejunum: 18% Ileum: 10%.	I/II: 44% III: 56%	78 (30/48)	No effect median DFS 48 vs. 53 months, (*p* = 0.41)	No effect median OS 59 vs. 64 months, (*p* = 0.57)
Huffman (2019) [32]	Retrospective single-center	Caucasian, US	Duodenum: 65% Jejunum 23% Ileum: 9% NS: 3%	I: 15% II: 41% III: 44%	241 (156/85)	N/A	Significant improvement for stage III with FOLFOX (*p* = 0.02).
Ning Li (2020) [33]	Retrospective	Asian, Chinese	Duodenum: 75.7% Jejunum: 4% Ileum: 14.9% NS: 5.4%	I: 30% II: 41% III: 29%	148 (93/55)	Significant improvement median DFS: 34 vs. 16 months (*p* = 0.002)	Significant improvement median OS: 40 vs. 26 months (*p* = 0.001)
Colina (2020) [26]	Retrospective multi-center	Caucasian, US	Duodenum: 52% Jejunum: 29% Ileum: 19%	I: 5% II: 45% III: 50%	257 (76/137)	No effect (*p* = 0.22)	No effect (*p* = 0.44)
Lee (2020) [29]	National Cancer database	Caucasian, US	“proximal” 53% “distal” 47%	I: 10.2% II: 36.8% III: 43.2% IV: 9.8%	7019 (not communicated)	N/A	Significant improvement for both proximal (*p* < 0.01) and distal (jejuno-ileal) tumors (*p* < 0.01)
Aparicio (2020) [3]	Prospective	Caucasian, French	Duodenum: 56.5% Jejunum: 24% Ileum: 19.5%	In situ: 2.5% I: 8.5% II: 33% III: 49.5% NS: 6.5%	179 (69/110)	N/A	No effect (*p* = 0.19)

CT: chemotherapy; DFS: disease-free survival; OS: overall survival; HR: hazard ratio; N/A: not applicable; ND: Not determined; NS: not specified; surg: surgery.

**Table 2:** Efficacy of adjuvant radio+/-chemotherapy versus surgery alone on disease-free survival and overall survival in small bowel cancer.

Author (Year)	Design	Population	Location	Stage	N (Surg/Surg + (C)RT)	DFS (Surg/Surg + (C)RT)	OS (Surg/Surg + (C)RT)
Bakaeen (2000) [28]	Retrospective single-center	Caucasian, US	Duodenum	0: 3% I: 25%% II: 37% III: 32% IV: 3%	67 (50/17)	N/A	No effect (*p* = 0.40)
Kim (2012) [49]	Retrospective single-center	Asian, Korea	Duodenum	I: 8.3% II:41.7% III:50%	24 (15/9)	5-year DFS rate: 64% vs. 80% (*p* = 0.42)	5-year OS rates: 30% vs. 47% (*p* = 0.38).
Kelsey (2007) [20]	Retrospective single-center	Caucasian, US	Duodenum	I: 19% II:56% III:13% IV: 6% NS: 6%	32 (16/16)	5 years DFS rate: 54% vs. 44% (*p* = 0.55)	5-year OS rates: 57% vs. 44% (*p* = 0.42).
Poultsides (2012) [50]	Retrospective single-center	Caucasian, US	Duodenum	I–II: 36.6% III: 63.4%	112 (78/34)	N/A	5-year OS rates: 47% vs. 48% (*p* = 0.82).

CT: chemotherapy; DFS: disease-free survival; OS: overall survival; CRT: chemoradiotherapy; DFS: disease-free survical; OS: overall survival; NS: not specified; surg: surgery.

**Table 3:** Prognostic factors for overall survival.

Author (Year)	Prognostic Factors				
	N+ vs. N0	Tumor Size (T4 vs. Other)	Grade (Poorly vs. Well/Moderate Differentiated)	Positive Resection Margin	Other
Overman (2010) [34]	Significant for lymph node ratio ≥ 10 *p* = 0.02 (multivariate)	NS (*p* = 0.14) (univariate)	Significant HR: 8.41 (1.79–39.54), *p* = 0.01 (multivariate)	N/A	N/A
Halfdanarson (2010) [36]	Significant for: -Lymph node ratio, ≥50% vs. <50%, *p* < 0.001. (univariate) -Stage III, *p* = 0.002 -Lymph node ratio, ≥50% vs. <50%, *p* = 0.03 (multivariate)	N/A	-Grades 3–4 vs. grades 1–2, *p* < 0.001 (univariate)	Significant for residual disease vs. no residual disease, *p* < 0.001 (univariate)	Significant for: -Age >60 y vs. ≤60 y, *p* < 0.005 -Male vs. female, *p* = 0.04 -Age, *p* = 0.008 (multivariate)
Dong Hoe Koo (2011) [37]	Significant *p* = 0.004 (multivariate)	NS *p* = 0.23 (univariate)	NS *p* = 0.32 (multivariate)	NS *p* = 0.58 (univariate)	N/A
Inoue (2012) [38]	NS *p* = 0.32 (univariate)	Significant for tumor size (mm) <70 vs. ≥70, p=0.0222 (multivariate)	N/A	N/A	Significant for location -Duodenum better prognosis vs. jejunum/ileum, *p* = 0.01 (multivariate)
Khurum Khan (2015) [39]	NS *p* = 0.26 (multivariate)	N/A	Significant *p* = 0.02 (multivariate)	N/A	Significant for: -LVI, *p* < 0.01 (multivariate)
Donat Duerr (2016) [40]	N/A	N/A	N/A	N/A	N/A
Aydin (2017) [41]	NS *p* = 0.93 (multivariate)	NS *p* = 0.12 (univariate)	NS *p* = 0.09 (univariate)	Significant HR: 0.16 (0.04-0.65), *p* < 0.01 (multivariate)	NS for -Perineural invasion, *p* = 0.31 -Vascular invasion, *p* = 0.25 (multivariate)
Eckert (2016) [35]	Significant HR: 1.81; *p* < 0.001 (multivariate)	Significant pT3-T4 vs. pT1-T2, *p* < 0.001 (multivariate)	Significant *p* < 0.001 (multivariate)	Significant *p* < 0.001 (multivariate)	Significant for: -Age ≤65 y vs. 66-74, *p* < 0.001 -Age ≤65 y vs. >75, *p* < 0.001 -Tumor location: duodenum vs. Ileum/jejunum, *p* = 0.01 -Surgery alone vs. adjuvant chemo, *p* < 0.001 (multivariate)
Huffmann (2019) [32]	Significant for: -Advanced N stage, higher lymph node ratio, *p* < 0.01 (univariate) -Lymphocyte-to-monocyte ratio, (univariate) *p* < 0.01	Significant for advanced T stage *p* = 0.04 (multivariate)	N/A	N/A	Significant for: -Male gender, *p* = 0.04 (univariate) -Age > 60 years, *p* = 0.02 (multivariate)
Ning Li (2020) [33]	NS *p* = 0.11 (multivariate)	NS *p* = 0.06 (univariate)	NS *p* = 0.42 (univariate)	N/A	Significant for: -Adjuvant chemotherapy, *p* < 0.01 (multivariate) -CA19-9 > 300, *p* < 0.01 (univariate) -«Symptoms» > 1 vs. 0–1, *p* = 0.06 (multivariate)
TIffany C lee, 2020 [29]	Significant *p* < 0.01 (multivariate)	Significant Tumor Size > 5 cm; *p* < 0.01 (multivariate)	Significant *p* < 0.01 (multivariate)	Significant -R1 vs. R0, *p* < 0.01 -R2 vs. R0, *p* < 0.01 (multivariate)	Significant for: -Male vs. female, *p* = 0.04 -“Distal” vs. “proximal”, *p* < 0.01 -Charlson-Deyo Comorbidity Score, *p* < 0.01 -Neoadjuvant chemo, *p* < 0.01 -Adjuvant chemo, *p* < 0.01 (multivariate)
Overman (2020) [26]	Significant *p* < 0.001 (multivariate)	Significant *p* = 0.01 (univariate)	Significant *p* = 0.01 (multivariate)	NS *p* = 0.11 (univariate)	Significant for MMR status *p* = 0.002 (univariate)
Aparicio (2020) [3]	Significant *p* = 0.01 (univariate)	Significant *p* < 0.01 (multivariate)	Significant *p* = 0.047 (multivariate)	N/A	N/A
Vanoli (2021) [65]	NA	Significant *p* = 0.049	NS *p* = 0.40	N/A	Mismatch repair deficiency (*p* = 0.019), glandular/medullary histologic subtype (*p* = 0.00), and celiac disease (*p* = 0.019) as significant predictors of favorable cancer-specific survival
Zhou (2021) [57]	NS *p* = 0.47 (multivariate)	NS *p* = 0.33 (multivariate)	N/A	N/A	Significant for location: better prognosis for -Jejunum, HR: 0.72 (0.63–0.82) -Ileum, HR: 0.70 (0.60–0.82) (multivariate)

NS: not specified, N/A: not applicable, LVI: lymphatic and venous invasion.

**Table 4:** Summary of international guidelines for localized small bowel adenocarcinoma surgical and adjuvant procedures.

Guidelines	Location	Surgical Procedures	Adjuvant Procedures
NCCN [8]	Duodenum	1st/2nd/4th portion of the duodenum: CDP or segmental resection with en bloc removal of regional lymph nodes or endoscopic resection 2nd portion of the duodenum: CDP with en bloc removal of regional lymph nodes	-T1T2N0M0/T3T4N0MO, (dMMR): observation -T3N0M0 (pMMR and no high risk feature): observation or 5FU/LV or capecitabine 6 mo -T3N0M0 (pMMR) with “high-risk” features or T4N0M0 (pMMR): observation or 5FU/LV or capécitabine 6 mo or FOLFOX/CAPOX 6 mo or chemoradiotherapy capecitabine-based (if positive margins) -Any N1N2: 5FU/LV or capécitabine 6 mo or FOLFOX/CAPOX 6 mo or chemoradiotherapy capecitabine-based (if positive margins)
	Jejunum-ileum	-Jejunum, proximal iléum: segmentectomy with en bloc removal of regional lymph nodes -Terminal ileum: Ileocolectomy with en bloc removal of regional lymph nodes	-T1T2N0M0/T3T4N0MO, (dMMR): observation -T3N0M0 (pMMR and no “high-risk” features): observation or 5FU/LV or capecitabine 6 mo -T3N0M0 (pMMR) with “high-risk” features or T4N0M0 (pMMR): observation or 5FU/LV or capécitabine 6 mo or FOLFOX/CAPOX 6 mo -Any N1N2: 5FU/LV or capécitabine 6 mo or FOLFOX/CAPOX 6 mo)
TNCD [2]	Duodenum	-CDP for tumors of the second portion of the duodenum and for proximal and distal infiltrating tumors (Grade C). Regional lymph node dissection must be performed, including the periduodenal and antero-posterior peripancreatic relays, hepatic relay of the right margin of the celiac trunk and the superior mesenteric artery. Extended lymph node dissection is not recommended (expert opinion). -Segmental duodenal resection is possible in cases of proximal (first portion of the duodenum) or distal tumors (a third portion of the duodenum, to the left of the superior mesenteric artery), non-infiltrating tumors, or tumors of the duodeno-Jejunal angle (expert opinion).	-Stage I: T1–2, N0, M0 Surgery only. -Stage II: T3, T4, N0, M0 Surgery only. OPTION: Adjuvant chemotherapy for T4 (expert agreement). CLINICAL TRIAL: PRODIGE 33-BALLAD stages I/II/III: Randomization between adjuvant chemotherapy (capecitabine/LV5FU2 or CAPOX/FOLFOX) versus observation. -Stage III: All T, N1–2, M0 NO RECOMMENDATIONS OPTIONS: Surgery followed by 6months of adjuvant chemotherapy with simplified FOLFOX4 or LV5FU2 or oral 5FU: capecitabine (expert agreement)
	Jejunum-ileum	-Segmental resection with lymph node dissection and jejuno-jejunal or ileo-ileal anastomosis (expert agreement). -For tumors involving the last ileal loop or the ileocecal valve, ileocecal resection or right hemicolectomy with resection of the ileal loop and ligature of the ileocolic artery at its origin, allowing the lymph node dissection (expert opinion).	-Stage I: T1–2, N0, M0: Surgery only. -Stage II: T3, T4, N0, M0: Surgery only. OPTION: Adjuvant chemotherapy for T4 (expert agreement). CLINICAL TRIAL: PRODIGE 33-BALLAD stages I/II/III: Randomization between adjuvant chemotherapy (capecitabine/LV5FU2 or CAPOX/FOLFOX) versus observation. -Stage III: All T, N1–2, M0 NO RECOMMENDATIONS OPTIONS: Surgery followed by 6 months of adjuvant chemotherapy with simplified FOLFOX4 or LV5FU2 or oral 5FU: capecitabine (expert agreement)

CDP: cephalic duodenopancreatectomy; Mo: months; NCCN: National Comprehensive Cancer Network; TNCD: Thésaurus National de Cancérologie Digestive. “High-risk” features according to NCCN guidelines in stage II SBA include close or positive resection margins, <5 lymph nodes examined of duodenal location or <8 lymph nodes examined if jejunal/ileal primary tumor location, and tumor perforation. Further consideration may be made for administering chemotherapy in patients with stage II disease who have a lymphovascular or perineural invasion or poorly differentiated histology due to data extrapolated from colorectal cancer studies.
